# A rare case of isolated duodenal metastases from hepatocellular carcinoma associated with p53 and ki-67 expression: a case report

**DOI:** 10.1186/1757-1626-2-9344

**Published:** 2009-12-17

**Authors:** Caroline Chung, Jaber Al Ali, David A Owen, Alan A Weiss, Eric M Yoshida, Isabella T Tai

**Affiliations:** 1 Radiation Oncology, BC Cancer Agency, 600 W10th Ave, Vancouver, V5Z 4E6, Canada; 2 Division of Gastroeneterology, Vancouver General Hospital, 899 12th Ave, Vancouver, V5Z 1M9, Canada; 3 Department of Pathology, Vancouver General Hospital, 899 12th Ave, Vancouver, V5Z 1M9, Canada; 4 Genome Sciences Centre, 675 W10th Ave, Vancouver, V5Z 1L3, Canada

## Abstract

Hepatocellular carcinoma (HCC) is the most common primary tumor of the liver worldwide. The incidence of HCC is increasing in North America secondary to rises in chronic liver disease from alcohol abuse and viral hepatitis. HCC most commonly metastasizes hematogenously or through lymphatics to the lungs and regional lymph nodes. Involvement of small bowel is rare and typically results from direct invasion and extension. We examined the molecular features related to this extremely rare case of isolated duodenal metastasis of HCC and noted p53 and Ki-67 positive staining. Here, we review the possible molecular and immunohistochemical studies that may aid definitive diagnosis and the evidence for the management of metastatic hepatocellular carcinoma.

## Background

Hepatocellular carcinoma (HCC) is the 6th most common cancer and the 3rd most common cause of cancer death worldwide [1]. It is most common in Asia and Africa; however the incidence of HCC in North America is rising, mostly related to cirrhosis from hepatitis C infection or alcohol abuse.

HCC most commonly metastasizes via blood vessels or lymphatics to the regional lymph nodes, lungs and bone. Other less common sites of spread that have been reported include the adrenal gland, stomach, peritoneum, kidney, spleen, heart, and brain. Metastasis to the small bowel is rare and the few cases of small bowel involvement have been reported only as case reports or case series. In most of the reported cases, small bowel involvement was located at the proximal duodenum as a result of direct extension from the primary tumor [2-6]. Hematogenous or lymphatic metastasis to the small bowel is incredibly rare.

The patient discussed in this case report presented with an upper gastrointestinal bleed secondary to HCC metastases located at the distal duodenum, which to our knowledge, has been reported only once previously[7] Here, we describe this rare condition, the challenges involved in clinical management, and the potential molecular markers associated with their aggressive behavior and poor clinical outcomes.

## Case presentation

This 53-year-old Canadian First Nations woman presented to emergency with abdominal pain, chest discomfort and melena. Her medical history included chronic hepatitis C, previous heavy alcohol consumption, cirrhosis and a recent diagnosis of multifocal hepatocellular carcinoma. Her other comorbid illnesses included type 2 diabetes mellitus, atrial fibrillation and valvular heart disease. At the time of initial diagnosis of HCC, a CT scan demonstrated multiple hepatic lesions and portal vein thrombosis. Although there was no evidence of tumor extension beyond the liver, her underlying heart disease and the presence of portal vein thrombosis deemed her an ineligible candidate for either surgical resection or transarterial chemoembolization. Since her diagnosis of HCC, she had been managed with long acting octreotide (Sandostatin, Novartis Canada, Dorval QC) 20 mg intramuscularly given monthly for seven months when she presented with abdominal discomfort and melena.

At presentation, she was hemodynamically stable and the physical examination was unremarkable apart from the presence of melena stool. Laboratory investigations showed hemoglobin 85 g/L, with MCV of 71.7 fl, and a normal serum AFP.

**Figure 1:**
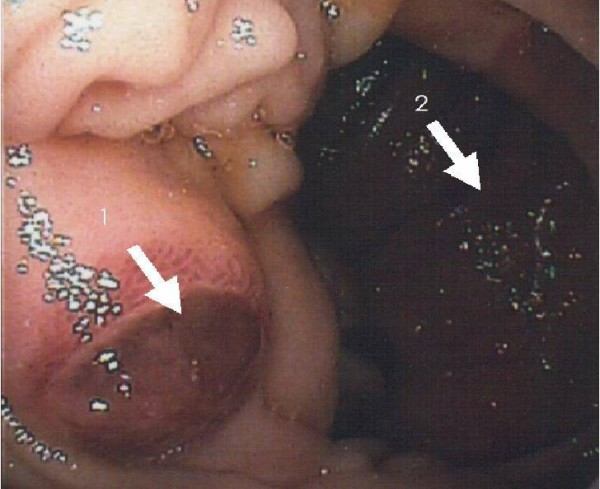
**Endoscopic photograph of metastatic lesions in duodenum at the end of arrows 1 and 2**.

Esophagogastroduodenoscopy (EGD) revealed two lesions in the third part of the duodenum: (i) a 2.5 cm soft nodule resembling the liver parenchyma with a dark red colour and hyperemic base, initially covered with a large clot (ii) a larger ulcerated lesion located distal to the first nodule, which was also covered in clot and more difficult to visualize (Figure 1). These lesions were not actively bleeding at the time of endoscopy, but the larger ulcerated lesion with overlying clot was injected with epinephrine to prevent further hemorrhage. There were no other obvious sources of bleeding and no identifiable esophageal or gastric varices. An EGD was repeated 48 hours later to reassess the duodenal lesions and multiple biopsies were taken at this repeat endoscopy. A repeat CT scan of the abdomen again revealed multifocal HCC and portal vein thrombosis, as seen at diagnosis, but no evidence of local invasion or extension into the duodenum.

## Pathological findings

**Figure 2:**
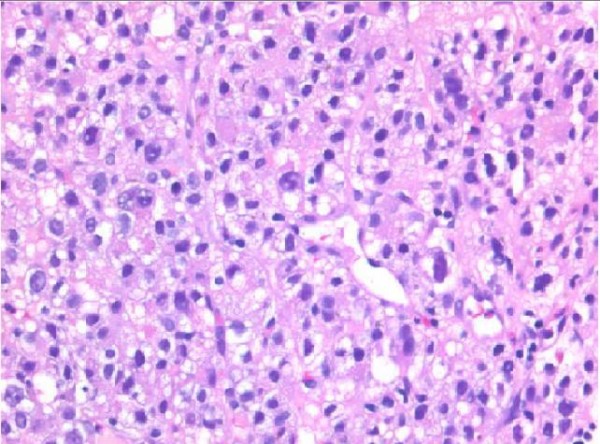
**Endoscopic biopsy specimen of duodenal lesion stained with hematoxylin and eosin**. The tissue has a sinusoidal arrangement of cells with large nuclei containing prominent nucleoli and eosinophilic cytoplasm. Low-grade nuclear pleomorphism is present (magnification × 200).

**Figure 3:**
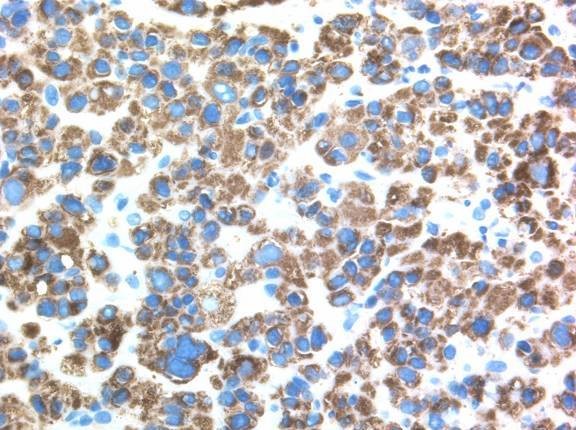
**Immunostain of the duodenal tumor showing diffuse cytoplasmic heppar 1 positivity**.

The biopsy fragments showed a moderately differentiated carcinoma growing in a sheet-like fashion with an indistinct sinusoidal pattern. The tumor cells had abundant pale pinkish or clear cytoplasm and vesicular nuclei with prominent nucleoli (Figure 2). Mucin production and bile secretion were both absent. Low-grade cytologic pleomorphism was present but there were only occasional mitotic figures. The histological appearances were considered typical for a moderately-differentiated hepatocellular carcinoma. Immunostaining was carried out and positive results were obtained for Heppar 1 (hepatocyte paraffin 1) (Figure 3) and interestingly, α-fetoprotein (AFP), confirming the diagnosis of metastatic hepatocellular carcinoma.

Ki-67 immunohistochemical staining was performed to assess proliferative activity in the tumor. One thousand cells were counted and 100 cells (10%) were positive. The expression of a tumor suppressor, p53, was also assessed. Twenty four percent of cell nuclei were either lightly or heavily stained.

## Discussion

Hematogenous metastasis to the duodenum from any primary malignancy is generally uncommon. Hematogenous metastasis from HCC to the duodenum is exceedingly rare. There have been several primary tumors with reported hematogenous metastases to the duodenum including carcinomas of the lung, breast, and malignant melanoma [8-10]. In these cases, regardless of the primary malignancy, diffuse and multiple metastases to several visceral sites (particularly lung and liver) were present when a duodenal metastasis was found, such that the duodenal involvement was of little clinical significance in the overall management. Isolated duodenal metastasis from HCC, as described in this case, has only been published once before. This rare presentation posed both a diagnostic and management challenge.

In this patient with known multifocal HCC, duodenal lesions resembling liver parenchyma were found endoscopically, raising the suspicion that these lesions were metastatic deposits of HCC. The majority of cases of duodenal involvement with HCC are a result of local extension of the primary tumour, but the CT scan demonstrated no local invasion into the small bowel [2,5,6]. If these lesions were considered metastatic deposits resulting from hematogenous spread to the duodenum, extrapolating from the available literature of other primary cancers, this would present in the setting of widespread metastatic disease [8-10]. However, there was no distant metastatic disease noted on the staging CT scan, particularly in the common sites of distant metastases: lung and bone.

In the setting of an unclear clinical presentation and small biopsy specimens, it is often necessary to confirm the histologic diagnosis using molecular and immunohistochemical studies. This is particularly true for endoscopically obtained biopsies, which are frequently small and often damaged with crush injury. Immunostaining of the biopsy fragments for Heppar 1 and AFP may help to resolve the histologic diagnosis of HCC. Heppar 1 immunostaining is present in 86% of hepatocellular carcinomas [11]. However, staining may also be present in 30-50% of gastrointestinal adenocarcinomas. Other intra-abdominal neoplasms may have similar histologic appearances to HCC with a sinusoidal growth pattern and possibly clear cell differentiation. For example renal cell carcinoma is generally negative for Heppar 1 staining [12]. Alpha-fetoprotein immunopositivity is encountered in only 37% of hepatocellular carcinomas but this is a more specific immunostain [13]. Adenocarcinomas arising from the lung and gastrointestinal tract are almost invariably AFP negative. The only other major class of neoplasms that are AFP positive is germ cell tumors, especially endodermal sinus tumors. These are usually histologically distinct and unlikely to be confused with a hepatocellular carcinoma. In our case, the combination of clinical knowledge of a multifocal primary HCC together with positive Heppar1 and AFP staining conferred the diagnosis of metastatic HCC to the small bowel.

The presence of high levels of p53 and Ki-67 expression in this case correlates with a more aggressive tumor phenotype. Positive p53 and Ki-67 markers (positive staining in >10% cells), in addition to high serum AFP levels, have been suggested as predictors of poor clinical outcomes and earlier recurrence of HCC [14]. Our findings support previous reports of these more aggressive HCC phenotypes that have metastasized to the small bowel.

With regards to treatment options, there is somewhat limited evidence to guide our management approaches, particularly for advanced tumour stages. Attempted treatments in the advanced setting include chemoembolisation, arterial or systemic chemotherapy, I^131 ^lipiodol, hormonal therapy, immunotherapy, and octreotide. Meta-analysis of the randomized trials comparing chemoembolisation with conservative management has shown a survival benefit with chemoembolisation. The chemotherapy agents previously tested for chemoembolisation include doxorubicin and cisplatin. On the other hand, meta-analysis of tamoxifen compared with conservative management failed to show a survival benefit [15]. A number of chemotherapy regimens have also been evaluated in the palliative setting with no particular single agent or combination chemotherapy regimen found to be particularly effective or superior.

In this case, the patient's management was somewhat limited by the presence of portal vein thrombosis, which made her ineligible for chemoembolization, and her other medical comorbidities, which would pose significant risk with more aggressive treatment. Given the available evidence around palliative single agent chemotherapy, she was treated with six cycles of doxorubicin every three weeks at disease progression. She survived another seven months on this regimen. It is difficult to know whether this treatment extended her survival in light of her extremely rare presentation of isolated duodenal metastasis.

## Conclusions

This is the second reported case of isolated duodenal metastasis of HCC without evidence of direct invasion or widespread disseminated metastases. Given this rare presentation, the diagnostic challenges were reviewed with descriptions of possible molecular and immunohistochemical studies that may help establish a definitive diagnosis. A brief review of the evidence for various treatment options in the setting of metastatic HCC was also presented, which supported the management of this particular patient. This case demonstrates the variability in presentation of metastatic HCC but also emphasizes the need for further tools to improve diagnosis and treatment of metastatic hepatocellular carcinoma.

## Abbreviations

AFP: alpha fetoprotein; CT: computed tomography; EGD: esophagogastroduodenoscopy; HCC: Hepatocellular carcinoma; VEGF: Vascular endothelial growth factor.

## Consent

Written informed consent was obtained from the patient for publication of this case report and any accompanying images. A copy of the written consent is available for review by the Editor-in-Chief of this journal.

## Competing interests

The authors declare that they have no competing interests.

## Authors' contributions

CC and JAA collected the information and wrote the case history of manuscript. CC finalized and submitted the manuscript. DO collected the pathological information and contributed to the pathological component of the case history and discussion of the manuscript. AW contributed to the endoscopic photos for the case history. EMY and IT contributed to the discussion and edited the final version of the manuscript. All authors read and approved the final manuscript.
